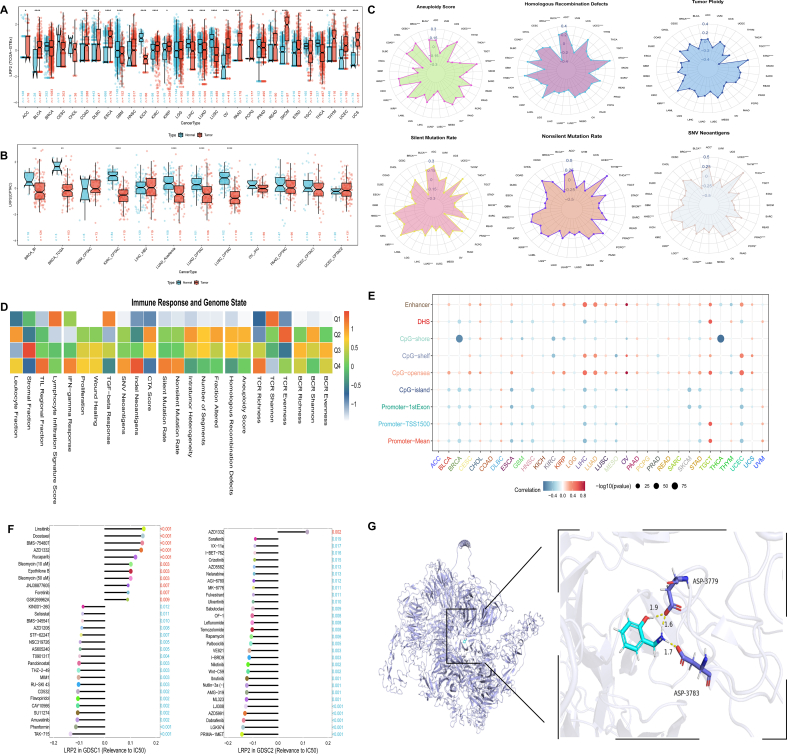# Ammonia-induced cell death patterns in glioblastoma: Immune landscape, prognostic signature, and therapeutic target identification

**DOI:** 10.1016/j.gendis.2025.101979

**Published:** 2025-12-12

**Authors:** Sheng-Qi Hu, Jing Wang, Jing Wang, Chun Zeng, Rudong Chen

**Affiliations:** a Department of Neurosurgery, Tongji Hospital, Tongji Medical College, Huazhong University of Science and Technology, Wuhan, Hubei 430030, China; b Beijing Neurosurgical Institute, Capital Medical University, Beijing 100070, China; c Department of Neurosurgery, Beijing TianTan Hospital, Capital Medical University, Beijing 100070, China; d Department of Neurosurgery, Peking University International Hospital, Beijing 100070, China; e China National Clinical Research Center for Neurological Diseases, Beijing 100070, China

Glioblastoma (GBM) remains one of the most lethal primary brain tumors due to its metabolic reprogramming and highly immunosuppressive microenvironment.^1^ Ammonia, a byproduct of glutamine metabolism, induces a form of regulated cell death termed ammonia-induced cell death (AICD), which was implicated in the impairment of immune cell function, particularly the survival of effector CD8^+^ T cells, through metabolic reprogramming during immune activation.^2^^,^^3^ This pathway has emerged as a critical mechanism by which GBM evades immune surveillance, contributing to its resistance to immunotherapies.^4^ However, the role of AICD in GBM has not been systematically elucidated. Here, we performed an integrative analysis of bulk and single-cell transcriptomic data to characterize AICD-related gene expression and immune context in GBM. We constructed and validated a six-gene prognostic model (*ENO2, GJB2, GRP, IL4I1, LRP2, and RPE65*) that stratifies patients by survival and immune features. Spatial and single cell RNA sequencing (scRNA-seq) analyses revealed that AICD activity is enriched in mural cells and macrophages. Among independent prognostic AICD-DEGs, we identified *LRP2* as a potential therapeutic target on the basis of its widespread expression and role in key biological processes (e.g., cell signaling, immune modulation, and tumor progression across cancers).^5^ Pan-cancer evaluation highlighted LRP2 as a promising immunometabolic target, and molecular docking identified 2-hydroxybenzylamine (2-HOBA) as a compound with strong binding to *LRP2*. Our findings provide a novel prognostic tool and identify *LRP2* as a candidate therapeutic target, potentially restoring immune function in GBM. A schematic workflow is shown in Figure 1, while all supporting results and methods are detailed in Supplementary Materials.

**Figure 1:**
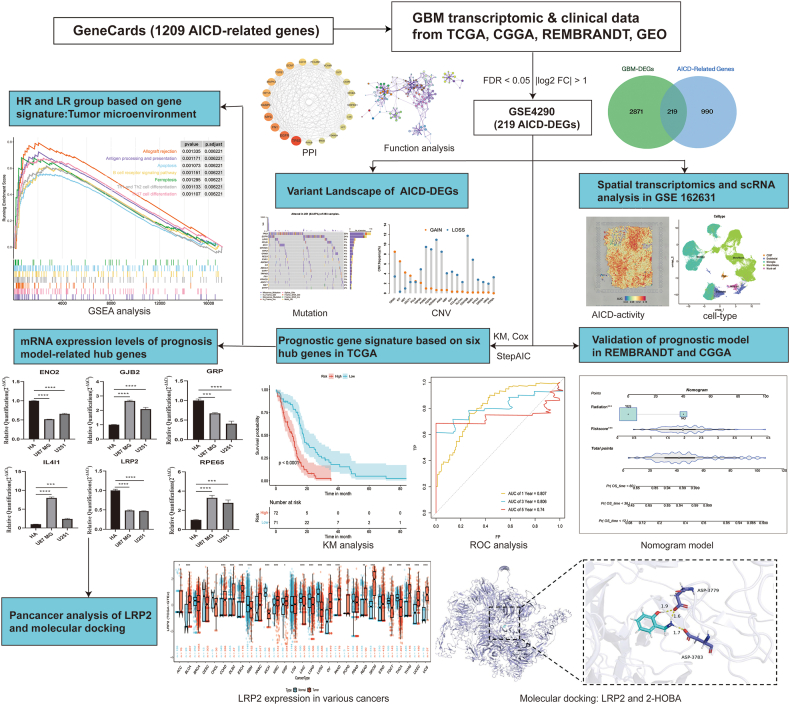
Flow chart of this research.

AICD-related genes were collected based on the GeneCards database with a relevance score threshold > 7 (Table S1). We identified 219 AICD-related differentially expressed genes (AICD-DEGs) in GBM compared to normal controls (Fig. S1A and S1B). Network analysis highlighted hub genes such as *TP53, EGFR, FN1, MYC,* and *CASP3* as key regulators (Fig. S1C). AICD-DEGs were frequently altered by missense mutations, and top five mutated genes were *TP53, EGFR, LRP2, PCLO,* and *IDH1* (Fig. S1D). Detailed patient-level mutation profiles are provided in Table S2. Copy number variation analysis showed significant alterations in the top 20 mutated AICD-DEGs (Fig. S1E). Functional enrichment analyses pointed to roles in carboxylic acid/Monocarboxylic acid/fatty acid metabolic acid metabolic process, xenobiotic metabolism, and nitrogen response (Fig. S1F).

Following SCTransform normalization, spatial transcriptomics data revealed five distinct cell types (CNS cells, endothelial cells, fibroblasts, macrophages, and tumor cells) (Fig. S2A and S2B). AUCell analysis showed that AICD activity was primarily enriched in tumor regions and correlated with specific cell content (Fig. S2C and S2D). To refine cellular resolution, scRNA-seq data from four GBM patients (96,318 cells) were integrated using Harmony, yielding 20 clusters that were classified into five major types: CD8^+^ T cells, endothelial cells, microglia, monocytes/macrophages, and mural cells (Fig. S2E and S2F). Cell–cell interaction mapping highlighted strong communication among microglia and monocytes/macrophages (Fig. S2G). Using ssGSEA, we quantified AICD-DEG expression across all cell types, revealing that monocytes/macrophages and mural cells exhibited the highest AICD activity, suggesting their prominent role in mediating AICD-associated immune modulation within the GBM microenvironment (Fig. S2H and S2I). To assess functional states, we performed M1/M2 scoring, which revealed significantly higher AICD activity in pro-tumoral M2-like macrophages than in anti-tumoral M1-like macrophages (Fig. S3A and S3B), linking AICD to immunosuppressive macrophage populations in GBM.

Univariate Cox regression identified 27 AICD-DEGs associated with GBM prognosis, which were refined to 14 genes via Kaplan–Meier analysis (Table S3 and S4). Stepwise multivariate regression yielded a six-gene signature (*ENO2*, *GJB2*, *GRP*, *IL4I1*, *LRP2*, and *RPE65*), forming the following risk score formula: Risk score = 0.3117 × *ENO2* + 0.2068 × *GJB2* + 0.3292 × *GRP* + 0.2965 × *IL4I1* − 0.4467 × *LRP2* − 0.2718 × *RPE65* (Fig. S4 and Table S5). Patients were stratified into high-risk (HR) and low-risk (LR) groups based on the median score. LR patients had significantly longer median overall survival in the TCGA cohort (17.7 *vs.* 10.6 months, *P* < 0.0001) (Fig. S5A). This prognostic value was validated in REMBRANDT (18.0 *vs.* 13.4 months, *P* = 0.0006), CGGA325 (16.1 *vs.* 9.5 months, *P* = 0.0018), and CGGA693 (13.4 *vs.* 11.5 months, *P* = 0.066) (Fig. S5B–S5D). The distribution of risk scores and survival status across all cohorts is illustrated in Supplementary Figure 5E–5H.

Univariate and multivariate Cox analyses confirmed that the AICD-based risk score was an independent prognostic factor in GBM, even after adjustment for clinical variables (Fig. S6A and S6B). In the TCGA cohort, both risk score and radiation status remained significant predictors of overall survival. We further examined correlations between the six-gene signature and clinical parameters, including survival time, Karnofsky performance score, age, gender, race, and treatment status (Fig. S6C). A prognostic nomogram integrating risk score and radiation was constructed (Fig. S6D). LR patients had significantly longer median survival than HR patients (17.7 *vs.* 10.4 months, *P* < 0.0001) (Fig. S6E). The nomogram achieved strong AUC values for 1-, 3-, and 5-year survival (0.807, 0.806, and 0.740, respectively) (Fig. S6F). Decision curve analysis indicated greater net clinical benefit at 3 and 5 years, supporting the model's utility in individualized prognosis (Fig. S6G).

CIBERSORT analysis revealed greater infiltration of M1 macrophages, resting dendritic cells, and neutrophils in the HR group compared to the LR group (Fig. S7A). Expression levels of the six prognostic genes were strongly correlated with these immune cell populations (Fig. S7B). ScRNA-seq further showed *IL4I1* to be enriched in monocytes/macrophages, while *ENO2* was localized to mural cells (Fig. S7C). ESTIMATE scores indicated a higher immune presence in HR patients (Fig. S7D). Drug sensitivity profiling revealed that LR patients were more responsive to doxorubicin, cisplatin, and etoposide (Fig. S7E). HR patients also exhibited higher hypoxia scores, suggesting a more immunosuppressive and treatment-resistant tumor microenvironment (Fig. S7F). Mutation analysis highlighted frequent alterations in *EGFR* and *MUC16* in both risk groups (Fig. S7G). GSEA demonstrated enrichment of fatty acid metabolism and DNA replication pathways in LR tumors, while HR tumors were associated with Th1/Th2 cell differentiation, apoptosis, and antigen presentation indicating divergent immune and metabolic landscapes between risk groups (Fig. S7H). We analyzed the expression of key T-cell exhaustion markers (e.g., *PDCD1*, *LAG3*, *CTLA4*, and *HAVCR2*) and immunosuppressive factors (*TGFB1* and *IL10*). The high-risk group showed significantly elevated expression of these genes, despite greater overall immune infiltration (Fig. S7I).

Quantitative RT-PCR analysis confirmed the expression patterns of the six AICD-related hub genes in glioma cell lines (Fig. S8A). *ENO2, GRP,* and *LRP2* were downregulated, while *GJB2, IL4I1*, and *RPE65* were upregulated compared to human astrocytes. These results were consistent with bioinformatics predictions, supporting the reliability of the prognostic model (Fig. S8B).

*LRP2* expression was elevated in several cancers, including adrenocortical carcinoma (ACC), bladder cancer (BLCA), colon adenocarcinoma (COAD), kidney renal clear cell carcinoma (KIRC), kidney renal papillary cell carcinoma (KIRP), ovarian cancer (OV), and uterine corpus endometrial carcinoma (UCEC) (Fig. S9A). LRP2 protein expression was substantially lower in KIRC and LUAD compared with the controls, and was insignificantly lower in GBM (Fig. S9B). Genomic correlation analyses revealed associations between LRP2 and metrics such as aneuploidy, homologous recombination deficiency, ploidy, mutation burden, and neoantigen load across cancers, though these were not significant in GBM (Fig. S9C). LRP2 expression was also linked to immune infiltration and DNA damage indicators, with cancer-type-specific variability in methylation levels, suggesting potential epigenetic regulation (Fig. S9D). A positive correlation between LRP2 and sensitivity to various chemotherapeutic agents highlights its therapeutic relevance (Fig. S8E). High-throughput virtual screening identified 2-HOBA as a top candidate, exhibiting strong binding affinity (−7.297 kcal/mol) and forming three hydrogen bonds with LRP2 residues Asp-3779 and Asp-3783, suggesting stable interaction and therapeutic potential in restoring LRP2 function (Fig. S9F and Table S6).

In summary, we comprehensively explored the link between AICD and GBM, integrating analyses of molecular pathways, single-cell mapping, immune infiltration, and clinical features. A prognostic signature comprising six AICD-DEGs (*ENO2*, *GJB2*, *GRP*, *IL4I1*, *LRP2*, and *RPE65*) demonstrated strong predictive accuracy. Additionally, 2-HOBA emerged as a potential lead compound for targeting LRP2 in GBM based on the computational screening.

## CRediT authorship contribution statement

**Sheng-Qi Hu:** Writing – original draft, Visualization, Methodology, Investigation, Formal analysis, Conceptualization. **Jing Wang:** Writing – original draft, Visualization, Methodology, Investigation, Formal analysis, Conceptualization. **Jing Wang:** Writing – original draft, Visualization, Methodology, Investigation, Formal analysis, Conceptualization. **Chun Zeng:** Writing – review & editing, Validation, Supervision, Conceptualization. **Rudong Chen:** Writing – review & editing, Validation, Supervision, Conceptualization.

## Conflict of interests

The authors have no conflict of interests to declare.

## Figures and Tables

**figs1:**
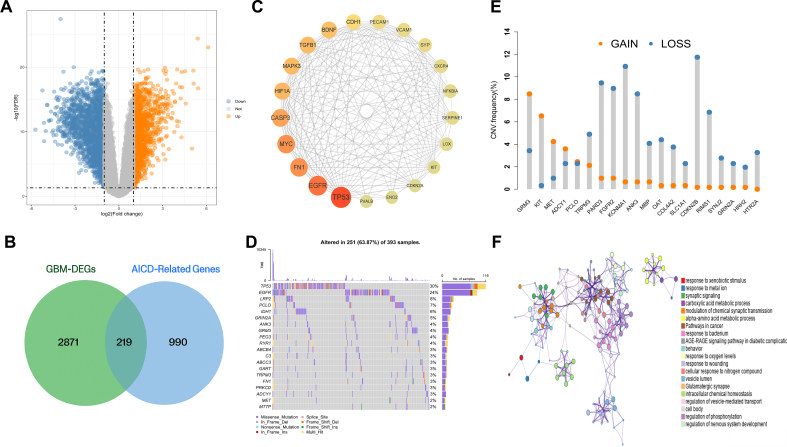


**figs2:**
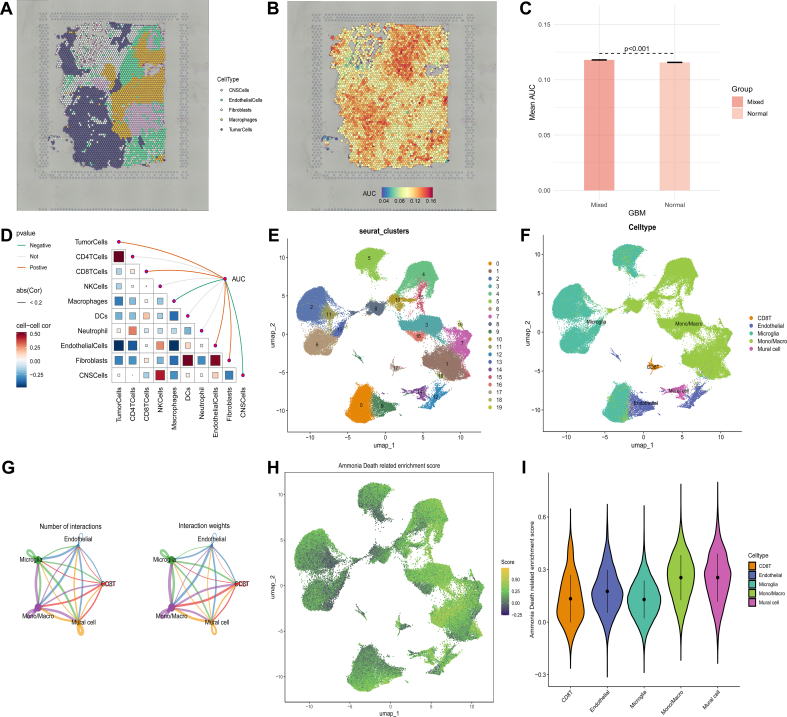


**figs3:**
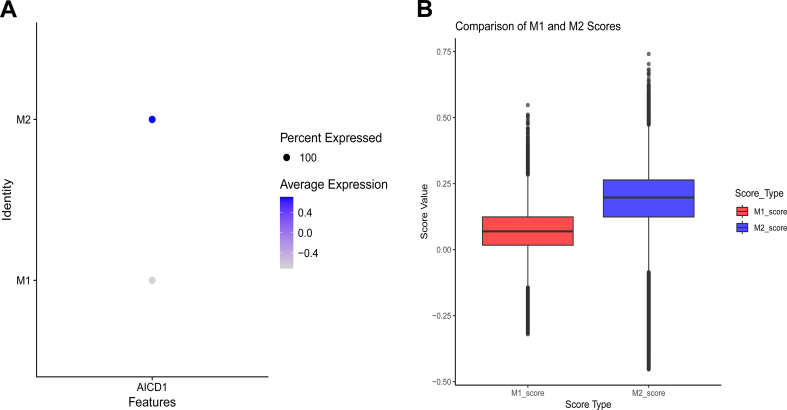


**figs4:**
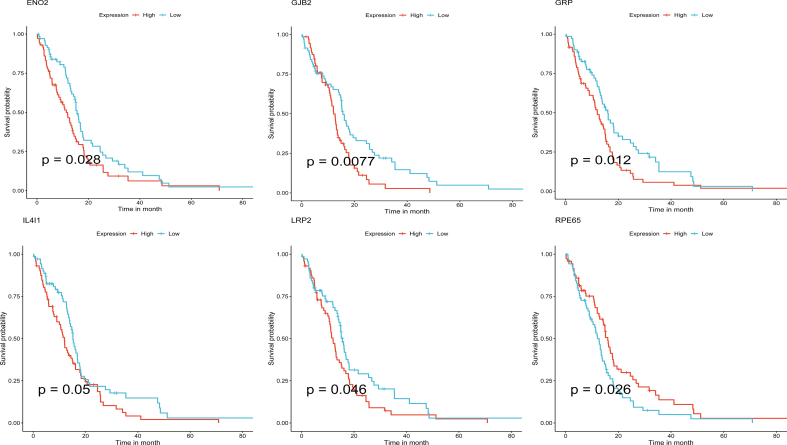


**figs5:**
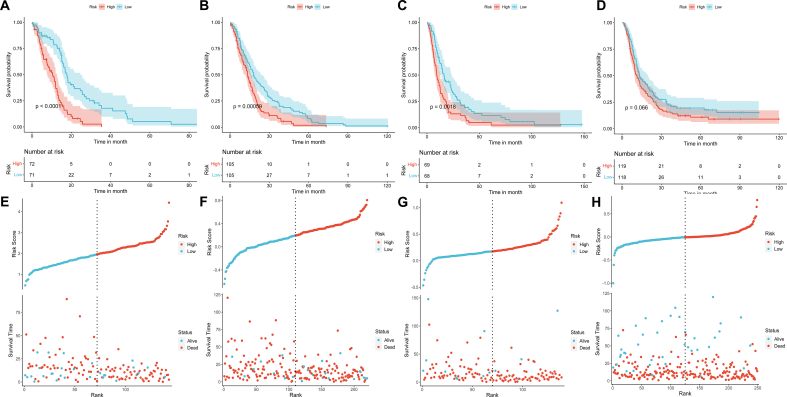


**figs6:**
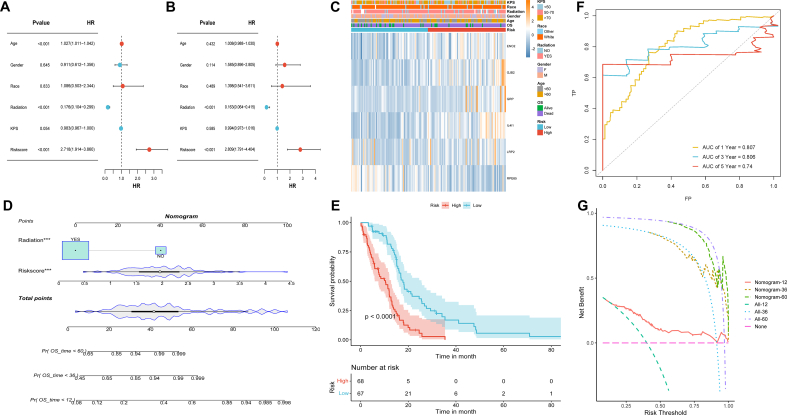


**figs7:**
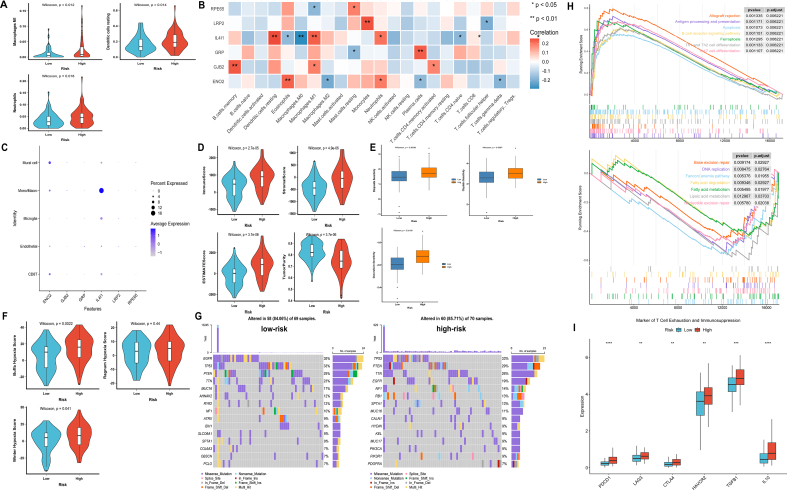


**figs8:**
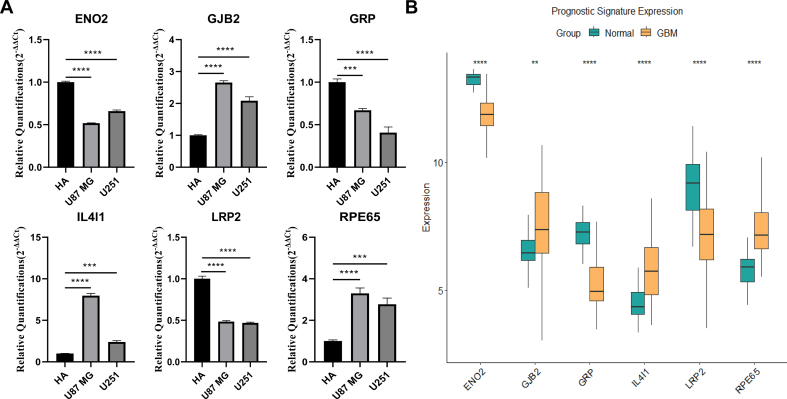


**figs9:**