# Supplementary material for: Ammonia-induced cell death patterns in glioblastoma: Immune landscape, prognostic signature, and therapeutic target identification
Source: Genes Dis. 2025 Dec 12;13(5):101979. doi: 10.1016/j.gendis.2025.101979 (PMC13194177; doi:10.1016/j.gendis.2025.101979)
Supplement: Multimedia component 1 [file mmc1.docx]

**Materials and methods**

**Data acquisition and preprocessing**

Bulk RNA sequencing data and clinical annotations for GBM patients were obtained from The Cancer Genome Atlas (TCGA) via the UCSC Xena project (https://gtexportal.org/home/) and used as the training cohort. A total of 143 GBM samples were included. For external validation of gene expression profiles and survival analyses, datasets from REMBRANDT (n = 210) were downloaded from the Gene Expression Omnibus (GEO) database (<http://www.ncbi.nlm.nih.gov/geo/>), while CGGA 325 (n = 137) and CGGA 693 (n = 237) were sourced from the Chinese Glioma Genome Atlas (CGGA) (http://www.cgga.org.cn). Differential expression analysis was conducted using the GSE4290 dataset (77 tumor and 23 normal samples) from GEO, with scRNA-seq data for human GBM (GSE162631) also acquired from GEO for further analysis. GBM spatial transcriptome data were sourced from the GBM Whole Transcriptome Analysis 10X platform. A total of 1,209 AICD related genes were compiled from the GeneCards database (https://www.genecards.org/), based on a relevance score threshold > 7, as detailed in Table S1.

**Processing of GBM spatial transcriptome data**

Spatial transcriptome sequencing data were processed using the R package Seurat. The workflow included normalizing unique molecular identifier counts, scaling the data, and identifying the most variable features using the SCTransform method. Principal component analysis (PCA) was performed to reduce the dimensionality of the data, followed by unsupervised clustering using the top 30 most significant principal components. The SpatialFeaturePlot function was used to visualize clusters, while the AUCell R package quantified AICD-related activities at spatial transcriptome resolution.

**scRNA-seq data analysis**

The 10X scRNA-seq data were processed and converted into a Seurat object using the Seurat R package. Cells with < 50 detected genes or > 5% mitochondrial gene expression were excluded. PCA was applied to the top 1,500 most variable genes. Cell clustering was performed using the FindNeighbors and FindClusters functions, considering the first 15 principal components. Marker genes for different clusters were identified using the FindAllMarkers function, applying a significance threshold of false discovery rate (FDR) < 0.01 and |log2 fold change (FC)| > 1. Cluster annotation was performed using the CellMarker 2.0 database. To quantify the expression of specific gene sets, the ssGSEA function within Seurat was used.

**Differential expression analysis and functional analysis**

Differential expression analysis was conducted using the limma R package to identify AICD-DEGs between GBM and normal samples in the GSE4290 dataset, applying a cutoff value of FDR < 0.05 and |log2 FC| > 1. Gene Ontology (GO) and Kyoto Encyclopedia of Genes and Genomes (KEGG) enrichment analyses were performed on AICD-DEGs using the Metascape tool (<https://metascape.org>), with an adjusted *P* value < 0.05 considered to indicate statistical significance. Somatic mutations in AICD-DEGs were detected using cbioportal ((http://www.cbioportal.org/), and the maftools R package. A protein–protein interaction (PPI) network for these genes was constructed using the STRING database.

**Development of an AICD scoring system**

Prognostic AICD-DEGs were identified via univariate Cox proportional hazards regression analysis with a significance threshold of *p* < 0.05. Kaplan-Meier (KM) survival analysis (*p* < 0.05) was performed to assess GBM prognosis, while stepwise model selection using the Akaike Information Criterion (stepAIC) in the MASS package was employed to identify the most significant AICD-DEGs associated with survival outcomes. These genes were incorporated into a multivariate Cox regression model, and a scoring system was developed by computing the linear combination of regression coefficients multiplied by normalized gene expression levels:

$$Risk score=\sum_{\mathcal{i}=1}^{N} (E\mathcal{x}p\mathcal{i}\times Coei)$$

Patients were categorized into high-risk (HR) and low-risk (LR) groups based on the median risk score. To evaluate the prognostic performance of the gene signature, KM and cumulative hazard curves were generated using the survminer and survival R packages.

**Construction of a prognostic nomogram model**

Univariate and multivariate Cox regression analyses were conducted to assess the prognostic independence of the AICD-related risk score and other clinical parameters. Using the rms and survivalROC packages, a nomogram was developed to estimate 1-year, 3-year, and 5-year mortality rates based on significant risk factors identified through Cox regression analysis. The accuracy of the nomogram was assessed using receiver operating characteristic (ROC) curves, decision curve analysis (DCA), and KM analysis.

**Assessment of immune characteristics**

Proportions of 22 immune cell types were estimated using the CIBERSORT R package. The maftools package was employed to create a waterfall plot illustrating the distribution of somatic mutations in GBM patients. The cBioPortal database (<http://www.cbioportal.org/>) was used to retrieve hypoxia scores for GBM. Drug sensitivity prediction was performed using the oncoPredict R package. Gene set enrichment analysis (GSEA) of KEGG pathways between the LR and HR groups was conducted using the clusterProfiler package, with a threshold for significantly enriched pathways set at *p* < 0.05.

**Pan-cancer analysis**

*LRP2* expression levels across multiple cancers were analyzed using the TCGAplot R package. The relationship between *LRP2* and chromosome scores were analyzed with aneuploidy score, homologous recombination deficit, ploidy score, non-silent mutation rate, and single-nucleotide variant neoantigen scores. Mutation data for *LRP2* across different cancers were analyzed using the cBioPortal tool. Correlation analyses were conducted to examine relationships between *LRP2* expression levels and chemotherapy drug sensitivity.

**Molecular docking**

The structures of active compound ingredients were sourced from the Compound Library and imported into ChemBio3D 14.0 for spatial conformation adjustments and energy optimization, saving the data in mol2 format. After processing with AutoDock Tools 1.5.6, the three-dimensional crystal structure of the target protein was downloaded from UniProt. Water molecules and organic molecules were removed using Notedad2. The target protein was preprocessed for molecular docking, including hydrogenation, charge distribution, and atomic type assignment. Docking simulations were conducted using AutoDock Vina, and the binding interactions were visualized with PyMOL 2.6.1.

**Cell lines and culture**

Human astrocytes were kindly provided by the Cell Bank of the Chinese Academy of Sciences. The U-87 MG and U251 MG glioblastoma cell lines were obtained from the Cell Resource Center of Peking Union Medical College and authenticated using short tandem repeat profiling. All cells were cultured in Dulbecco's modified Eagle medium (Gibco, USA) containing 10% fetal bovine serum (Gibco, USA) and were incubated at 37°C in 5% CO_2_.

**Quantitative real-time reverse transcription polymerase chain reaction (qRT-PCR)**

Total cellular RNA was isolated using TRIzol reagent (BS258A, Biosharp, Beijing, China), in accordance with the manufacturer's instructions. The purity and concentration of RNA were evaluated using a NanoDrop One Spectrophotometer (Thermo Fisher Scientific, USA). Total RNA (1 µg) was reverse transcribed into cDNA using All-in-One First-Strand Synthesis MasterMix (F0202, LabLead, Beijing, Chi126na), in accordance with the standard protocol. The following primers were generated against the indicated human genes:

*ENO2*, forward 5'-AGCCTCTACGGGCATCTATGA' and reverse 5'-TTCTCAGTCCCATCCAACTCC-3';

GJB2, forward 5'-TCGCATTATGATCCTCGTTGTG-3' and reverse 5'-GGGGAAGTAGTGATCGTAGCAC-3'; *GRP*, forward 5'-AAAGAGCACAGGGGAGTCTTC-3' and reverse 5'-TCCTTTGCTTCTATGAGACCCA-3'; *IL4I1*, forward 5'-GCCAAGACCCCTTCGAGAAAT-3' and reverse 5'-CCGATCCTGTTATCTGCCTCC-3'; *LRP2*, forward 5'-GGCCTGCTATAACACCAGTCA-3' and reverse 5'-ACTCATTGTGCAAGCATATCTCA-3'; *RPE65*, forward 5'-TTGGATCTGAGCCATTTTACCAC-3' and reverse 5'-GTCAGTAACCTCTACTCCTCGAA-3'.

qRT-PCR was performed on triplicate samples using a QuantStudio 5 (Applied Biosystems, USA) with 2× RealStar Fast SYBR qPCR Mix (A304,GenStar, Beijing, China). The β-actin gene (*ACTB*) was used as the internal control. Relative gene expression levels were calculated using the 2−ΔΔCt method.

**Statistical analysis**
Some statistical analyses were performed in R software (v4.3.1), namely Wilcoxon and Kruskal-Wallis tests for pairwise and multiple group comparisons, respectively, as well as KM curves and log-rank tests for survival analysis. The qRT-PCR data were analyzed using unpaired two-tailed t-tests in GraphPad Prism 8.0 software. Experimental data are presented as means ± standard deviation and are derived from at least three independent replicate experiments. A *p* value < 0.05 was considered statistically significant.

**Supplementary Tables and Figures**

**Supplementary Table 1 (Table S1)**: Ammonia-induced cell death related genes.

**Supplementary Table 2 (Table S2):** Patient-level mutation profiles.

**Supplementary Table 3 (Table S3):** Twenty-seven ammonia induced cell death differentially expressed genes were identified by the univariate Cox regression analysis.

**Supplementary Table 4 (Table S4):** Fourteen prognostic genes were identified by Kaplan-Meier survival analysis.

**Supplementary Table 5 (Table S5)**: Six hub genes were identified by multivariate cox analysis.

**Supplementary Table 6 (Table S6):** The top 20 compounds interacting with LRP2 were identified by high-throughput virtual screening of the Chinese compound library.

**Supplementary Figure 1 (Figure S1):** Variant landscape of AICD related genes in GBM patients. (A) Volcano plot of the DEGs in GBM (blue: down-regulated DEGs; orange: up-regulated DEGs; grey: unchanged genes), FDR < 0.05 and |log2FC| > 1. (B) Venn diagram between GBM-DEGs and AICD related genes. (C) The PPI network of the AICD-DEGs associated proteins. (D) An oncoplot of the molecular alteration landscape of AICD-DEGs in GBM. (E) The top 20 mutated AICD-DEGs exhibited significant CNV alterations. (F) GO and KEGG enrichment analyses of AICD-DEGs in GBM. Abbreviations: AICD, ammonia-induced cell death; GBM, Glioblastoma multiforme; DEGs, differentially expressed genes; FDR, False Discovery Rate; PPI, protein-protein interaction; CNV, copy number variations; GO, gene ontology; KEGG, Kyoto Encyclopedia of Genes and Genomes.

**Supplementary Figure 2 (Figure S2)**: Immunogenic AICD characteristics in spatial and single-cell transcriptomes. (A) Spatial transcriptomics data of GBM. (B) Spatial visualization of AICD intensity. (C) Differential anlysis of AICD related activity in mixed and normal regions. (D) Spearman correlation of AICD related activity with microenvironmental components at spatial transcriptome resolution. (E-F) Single cell types identified by marker genes. (G) Interaction analysis of cell types in the GBM sample, showing interaction number and strength. (H) The AICD related enrichment score (activity) in each cell. (I) The distribution of AICD related score in different cell types. Abbreviations: AICD, ammonia-induced cell death; GBM, Glioblastoma multiforme.

**Supplementary Figure 3 (Figure S3):** Comparison of ammonia-induced cell death (AICD) in M1/M2 score. A, A scatter plot showing the expression of the AICD across M1 and M2 subtypes. B, A box plot comparing the M1 (red) and M2 (blue) scores.

**Supplementary Figure 4 (Figure S4):** Kaplan-Meier survival analysis of six hub genes.

**Supplementary Figure 5 (Figure S5)**: Construction of a AICD related prognostic signature for GBM patients. (A-D) Overall survival in the low- and high-risk score group patients in TCGA-GBM, REMBRANDT, CGGA325 and CGGA693. (E-H) The distribution of risk scores and survival status in low- and high-risk score group in the TCGA, CGGA325, and CGGA693 cohorts. Abbreviations: AICD, ammonia-induced cell death; GBM, Glioblastoma multiforme; TCGA, The Cancer Genome Atlas; CGGA, Chinese Glioma Genome Atlas.

**Supplementary Figure 6 (Figure S6)**: Establishment and assessment of the nomogram survival model. (A) Univariate analysis for the clinicopathologic characteristics and risk score in TCGA-GBM. (B) Multivariate analysis for the clinicopathologic characteristics and risk score in TCGA-GBM. (C) The heatmap showing the relationship between gene expression levels and clinical characteristics. (D) A nomogram was established to predict the prognostic of GBM patients. (E) Kaplan-Meier analyses for the two GBM groups based on the nomogram score. (F) Receiver operating characteristic curve of the combined model for 1-, 3- and 5-year survival in TCGA-GBM. (G) Decision curve analysis of nomogram predicting 1-, 3-, and 5-year overall survival. Abbreviations: GBM, Glioblastoma multiforme; TCGA, The Cancer Genome Atlas.

**Supplementary Figure 7 (Figure S7)**: Analysis of tumor microenvironment of AICD-related prognostic signature. (A) Violin plot of Immune cell infiltration levels in LR and HR group. (B) Heatmap plot showing the expression levels of the six genes included in the prognostic were highly correlated with the proportions of these tumor-infiltrating immune cells. (C) Dot plot visualization showing the gene expression distribution across different cell populations. (D) Violin plot of Immune scores in LR and HR groups. (E) Drug sensitivity to doxorubicin, cisplatin, and etoposide in LR and HR groups. (F) Violin plot of hypoxia scores in LR and HR groups. (G) Oncoplot of difference analysis of molecular mutation in HR and LR groups. (H) GSEA enrichment analysis of LR and HR patients. I, Expression of T-cell exhaustion markers and immunosuppression markers in HR and LR patients. Abbreviations: AICD, ammonia-induced cell death; LR, low-risk; HR, high-risk.

**Supplementary Figure 8 (Figure S8)**: The mRNA expression levels of prognosis model related hub genes. (A) RT-qPCR assay displaying the different mRNA expression of ENO2, GJB2, GRP, IL4I1, LRP2, and SYN1 in HA cells and GBM cells. (B) The boxplot of bioinformatic results. Statistical differences between the groups were analyzed using unpaired two-tailed t-tests (* p < 0.05; ** p < 0.01; *** p < 0.001; **** p < 0.0001). All data are presented as the mean ± standard deviation (SD) and represented at least three independent replicate experiments.

**Supplementary Figure 9 (Figure S9)**: Pan-cancer analysis of LRP2 expression and molecular docking. (A) LRP2 mRNA expression across various cancers. (B) LRP2 protein expression across various cancers. (C) Radar plots representing the relationship of LRP2 with aneuploidy score, homologous recombination deficit, ploidy score, non-silent mutation rate, and single-nucleotide variant neoantigen scores across various cancers. (D) Heatmap showing the relationship between LRP2, immune infiltration, and genomic status. (E) Dot plot showing methylation levels of LRP2 across different genetic loci in various cancers. (F) Drug sensitivity analysis showing chemotherapy drug sensitivity across multiple cancers. (G) Molecular docking showing that 2-HOBA forms three hydrogen bonds with key LRP2 amino acid residues Asp-3779 and Asp-3783. Abbreviations: ACC, adrenocortical carcinoma; BLCA, bladder urothelial carcinoma; BRCA, breast invasive carcinoma; CESC, cervical squamous cell carcinoma and endocervical adenocarcinoma; CHOL, Cholangiocarcinoma; COAD, colon adenocarcinoma; DLBC, diffuse Large B-cell lymphoma; ESCA, esophageal carcinoma; GBM, glioblastoma multiforme; HNSC, head and neck squamous cell carcinoma; KICH, kidney chromophobe; KIRC, kidney renal clear cell carcinoma; KIRP, kidney renal papillary cell carcinoma; LGG, lower grade glioma; LIHC, liver hepatocellular carcinoma; LUAD, lung adenocarcinoma; LUSC, lung squamous cell carcinoma; OV, ovarian serous cystadenocarcinoma; PAAD, pancreatic adenocarcinoma; PCPG, pheochromocytoma and paraganglioma; PRAD, prostate adenocarcinoma, READ, rectum adenocarcinoma; SKCM, skin cutaneous melanoma; STAD, stomach adenocarcinoma; TGCT, testicular germ cell tumors; THCA, thyroid carcinoma; THYM, thymoma; UCEC, uterine corpus endometrial carcinoma; UCS, uterine carcinosarcoma.
